# Awareness of Memory Deficits in Early Stage Huntington's Disease

**DOI:** 10.1371/journal.pone.0061676

**Published:** 2013-04-19

**Authors:** Laurent Cleret de Langavant, Gilles Fénelon, Sarah Benisty, Marie-Françoise Boissé, Charlotte Jacquemot, Anne-Catherine Bachoud-Lévi

**Affiliations:** 1 INSERM U955 E01, Neuropsychologie Interventionnelle, Créteil, France; 2 Département d'Etudes Cognitives, Ecole Normale Supérieure (ENS), Paris, France; 3 AP-HP, Centre de Référence – Maladie de Huntington, Hôpital H. Mondor - A. Chenevier, Créteil, France; 4 Université Paris Est, Faculté de Médecine, Créteil, France; Federal University of Rio de Janeiro, Brazil

## Abstract

Patients with Huntington's disease (HD) are often described as unaware of their motor symptoms, their behavioral disorders or their cognitive deficits, including memory. Nevertheless, because patients with Parkinson's disease (PD) remain aware of their memory deficits despite striatal dysfunction, we hypothesize that early stage HD patients in whom degeneration predominates in the striatum can accurately judge their own memory disorders whereas more advanced patients cannot. In order to test our hypothesis, we compared subjective questionnaires of memory deficits (in HD patients and in their proxies) and objective measures of memory dysfunction in patients. Forty-six patients with manifest HD attending the out-patient department of the French National Reference Center for HD and thirty-three proxies were enrolled. We found that HD patients at an early stage of the disease (Stage 1) were more accurate than their proxies at evaluating their own memory deficits, independently from their depression level. The proxies were more influenced by patients' functional decline rather than by patients' memory deficits. Patients with moderate disease (Stage 2) misestimated their memory deficits compared to their proxies, whose judgment was nonetheless influenced by the severity of both functional decline and depression. Contrasting subjective memory ratings from the patients and their objective memory performance, we demonstrate that although HD patients are often reported to be unaware of their neurological, cognitive and behavioral symptoms, it is not the case for memory deficits at an early stage. Loss of awareness of memory deficits in HD is associated with the severity of the disease in terms of CAG repeats, functional decline, motor dysfunction and cognitive impairment, including memory deficits and executive dysfunction.

## Introduction

Huntington's disease is an inherited neurodegenerative disease in which patients suffer from behavioral, motor and cognitive disorders. In particular, their memory is impaired, showing poor retrieval capacity [1]. HD patients have been described as unaware of their motor symptoms, their behavioral disorders, their cognitive deficits [2], [3], [4], [5], [6], [7], [8], but also recently their memory deficits [9]. Although unawareness of memory deficits is a classical clinical feature in patients with Alzheimer's disease (AD) [10], [11], it was recently shown that patients with Parkinson's disease (PD) can report their own memory difficulties through auto-questionnaires [12]. Given that both PD and HD are basal ganglia neurodegenerative disorders yielding executive dysfunction and memory retrieval deficits [13], preserved vs. impaired awareness for memory deficits in PD [12] vs. in HD [9] respectively would suggest that unawareness for memory deficit might not depend on striatal dysfunction *per se* but on another neural basis. If this hypothesis were true, one would expect good awareness of memory deficits at early stage Huntington's disease where degeneration predominates in the striatum [14], [15] and unawareness of these deficits at more advanced stages. However the only study exploring awareness of memory deficits in HD [9] included patients with different levels of independence (from 60 to 100% on the independence scale [16]) (Sitek et al., personal communication) suggesting mild to moderately severe stages of the disease [17].

In neurological disorders, subjective auto-questionnaires have proven useful in the aim to evaluate patients' perception of their own subtle deficits [18], to appreciate their mood disorders [19] or to have an insight into their quality of life after medical interventions [20]. This approach does not force the patient to turn to a neurologist for the follow-up of the disease and presumably guarantees an ecological evaluation of the symptoms. However, subjective evaluation of deficits does not appear to be a reliable methodology for patients who are not aware of the presence of or the severity of these symptoms, as might be the case in HD [2], [3], [4], [5], [6], [7], [8], [9]. In order to check if patients suffer from unawareness of a particular deficit, one can compare the subjective evaluation achieved by patients to the one completed by an external rater close to the patient, such as a relative. However, even if proxies are privileged observers of patients' behavior and performance in naturalistic environment [3], potential motivational and affective biases may affect their evaluation of patients' deficits [11]. Another possibility is to compare a subjective evaluation by the patient with an objective measure of the deficit, using standardized scales or tools. Yet, one can discuss the ecological validity of these scales. Therefore, combining both of the above strategies to ascertain patient's deficit awareness appears most appropriate.

The goal of the present study is to assess awareness of memory deficits in HD patients and in their proxies, especially at early stage of the disease. We compared subjective ratings of memory deficits assessed by patients and proxies with the objective performance of patients at a comprehensive neuropsychological battery including memory tests [21]. We used a subjective memory questionnaire [22] already used in AD [10], PD [12] and HD [9]. In order to test whether the accuracy of these evaluations was influenced by the evolution of the disease or by specific cognitive impairments, we enrolled both mildly impaired (Stage 1) and moderately impaired (Stage 2) HD patients. Hence, our protocol had the ability 1) to investigate awareness of memory deficits in HD at early stage, and 2) to question the validity of proxies' subjective evaluation of patients' memory deficits.

## Methods

### Patients

Forty-six consecutive patients with manifest HD (15 women, 31 men; mean age 42.5 years, range 21–58 years; mean 12.2 years of education, range 7–20) participated in the study. The patients were all genetically confirmed for HD (mean CAG repeats = 46.2±SD 4.8). Their mean Total Functional Capacity Scale score (TFC) [23] was 10.5 (range 7–13), indicating mild to moderate impairment of autonomy. Twenty five patients were at Stage 1, (HD1), and 21 at Stage 2 according to Shoulson's classification (HD2) [23]. When available, proxies exempt from the Huntingtin gene were enrolled (N = 33); proxies were related to 19 patients at Stage 1 (HD1Prox) and to 14 at Stage 2 (HD2Prox). Among proxies, there were 18 spouses, 13 family relatives and 2 close friends. The patients and proxies responded to the subjective memory questionnaires independently and prior to the cognitive testing of the patients. This study was part of a cohort study (RHLF) which was approved by the local ethics committee (Comité de Protection des Personnes de l'hôpital Henri Mondor, Créteil, France). Written informed consent was obtained from all patients after the study had been fully explained to them. Proxies provided a consent taking the form of a non-opposition to participate to the study in agreement with the French regulation law. This research was conducted in France.

### Subjective memory questionnaire

Patients were asked to rate their own memory deficits and proxies were asked to rate the patients' memory deficits using a French adaptation of the subjective memory questionnaire [10]. In the proxy's version, we replaced the personal pronoun “I” by “he” or “she” as appropriate. This questionnaire consisted of 20 items evaluating memory in comparison to the premorbid period [10]. Ratings were performed on a 9-point scale (from −4 to +4), yielding a total score ranging from −80 to +80. A negative score indicated that the participant reported a deterioration of memory, a positive score its improvement. Subscores for various memory components were obtained by pooling the corresponding items: global evaluation of memory, attention, retention, recall, remote memory for personal events and metamemory [10], the latter component corresponding to the ability to make second-order judgments about one's own memory (“If I were asked a month from now, my ability to remember this questionnaire would be…”).

### Patient assessment

Neurological examination used the Unified Huntington's Disease Rating Scale (UHDRS) (motor, cognitive and functional part) [16]. Depression was assessed using the Montgomery and Åsberg Depression rating scale (MADRS) [24]. Neuropsychological examination included general evaluation (Mattis Dementia-Rating scale (MDRS) [25]) and specific evaluations of attention and executive functions (Trail Making Test form A (TMT A) and B (TMT B), the Stroop Color Interference Test (Stroop C/W), the Wisconsin modified card sorting test (MCST) [26] and both literal (P and M) and categorical (animals) fluency collected in two minutes). Memory was assessed through the immediate and delayed recall after 20 minutes of the French adaptation of the Free and Cued Selective Reminding Test (FCSRT) [27], [28] and with the immediate recall and recognition of the Rey Auditory Verbal Learning test (RAVLT) [29]. The delayed recall in the RAVLT was not included in order to avoid confusion with the delayed recall of the FCSRT. Although the RAVLT has been used in similar studies evaluating awareness of memory deficits in PD [12], [30] and HD [9], the FCSRT might be a more appropriate test for objective evaluation of memory deficits in HD. Indeed, the FCSRT better controls the encoding process than the RAVLT [27], [31], which warrants better accuracy in patients with attention and executive dysfunction as it is the case in HD.

### Awareness indexes

In order to explore patients' awareness of their memory symptoms, we calculated three awareness indexes.

The first index compares subjective ratings in both patients and proxies. For each patient/proxy couple, the Δ(subjHD-subjProx) index was the result of subtracting the subjective rating by the proxy (subjProx) from the subjective rating by the patient (subjHD). A Δ(subjHD-subjProx) index lower than −25 suggests severe unawareness of memory deficits in the patient; an index greater than −5 suggests spared awareness [10].

The second index compares subjective ratings in patients to patients' objective memory performance [11], [32]. For objective performance (objHD), we calculated the mean z-score of the total free recall of FCSRT and the total recall of RAVLT; this data was available for all patients. Similarly z-scores were calculated for subjective memory ratings in patients (subjHD). The subtraction of the objective mean z-score from the subjective z-score (Δ(subjHD-objHD) index) indicates the degree of awareness of memory deficits for each patient. A negative index suggests spared awareness; a positive index suggests unawareness.

The third index similarly assesses proxies' awareness of patients' deficits, by replacing patients' subjective ratings by proxies' subjective ratings, yielding the Δ(subjProx-objHD) index.

### Statistical analysis

Two separate analyses were performed: first with the whole cohort of 46 patients, allowing stratification into stages and correlation with the disease severity, then with the subgroup of the 33 patients having available proxies, thus allowing the evaluation of the accuracy of subjective evaluation in both populations. We compared the data from the 46 patients with those from the subgroup of 33 patients by Welch's two-sample t-tests. Non-parametric Spearman correlations were run first between objective memory performance and subjective ratings, and second between clinical variables and awareness indexes. All analyses were performed using the 2.9.2 release of the R software [33]. Bonferroni corrections for multiple comparisons were not applied in this study because of the limited number of patients, as was also the case in previous explorations of unawareness of deficits in HD [2], [3], [4], [5], [6], [7], [8], [9].

## Results

### Patient assessment

HD1 and HD2 performances are displayed in Table 1. Forty-four percent HD1 and 90% HD2 were cognitively impaired with respect to their MDRS score. Memory performance remained normal on average for HD1 but not for HD2, who showed poor retrieval. Fifty-two percent HD2 and 28% HD1 were depressed according to the MADRS.

**Table 1:** Demographic characteristics and global assessment of the HD patients.

Measures, mean (SD; range)	HD1 (N = 25)	HD2 (N = 21)	Cut-off*	p
**Age**	43.1 (6.4; 30–56)	41.7 (9.6; 21–58)	-	ns
**Sex ratio M/F**	14/11	17/4	-	-
**Years of education**	13.3 (3.8; 7–20)	11.2 (2.7; 8–17)	-	0.06
**CAG repeats**	45.5 (4.4; 40–58)	46.9 (5.2; 41–61)	-	ns
**Disease duration**	3.7 (2.4; 0–9)	5.9 (1.5; 4–8)	-	<0.01
**Functional Decline-TFC**	11.9 (0.9; 11–13)	8.9 (1; 7–10)	-	<0.001
**UHDRS-Aptitude**	25.8 (1.1; 24–28)^a^	29.8 (3; 25–39)^b^	-	<0.001
**UHDRS-Motor**	24.8 (14.7; 0–47)^a^	40.5 (14.6; 18–78)^b^	>5	<0.001
**UHDRS-Psychiatric**	10.4 (10; 0–35)^a^	12.9 (11.4; 0–33)^b^	-	<0.001
**Global Cognitive Efficiency - MDRS**	133.7 (10.8; 93–144)	122.3 (11.4; 97–140)	≥136	<0.01
**Memory**				
**FCSRT**				
Total Free Recall	29.4 (7.8; 13–44)	19.5 (5.8; 8–27)	≥26	<0.001
Total Free & Cued Recall	44.8 (4.3; 30–48)	38.2 (7.5; 21–48)	≥37	<0.01
Recognition	15.8 (0.8; 12–16)	15.1 (1.1; 13–16)	-	<0.05
Delayed Free Recall	11.1 (3.2; 2–16)^c^	7.8 (3.3; 2–14)^d^	-	<0.01
Delayed Free & Cued Recall	15.3 (2.1; 7–16)^c^	13.4 (2.7; 9–16)^d^	-	<0.05
**RAVLT**				
Total Recall	42.4 (11.8; 16–61)	25.9 (9.1; 11–43)	≥40	<0.001
Recognition	13.5 (2.2; 6–15)	9.9 (3.8; 1–14)	-	<0.001
**Executive function**				
TMT A time	57.2 (25; 24–118)	94.5 (36; 38–164)	≤54	<0.001
TMT B time	145.7 (76; 52–240)	198.6 (52; 90–240)	≤135	<0.01
TMT B points	23.5 (3.5; 12–25)	18.3 (7.7; 2–25)	-	<0.01
Stroop C/W	32.7 (8.6; 19–51)	22 (10.8; 4–42)	≥35	<0.001
Literal fluency (P, M)	17.1 (8.1; 2.5–31.5)	10.1 (5.4; 2.5–20.5)	≥12.1	<0.01
Categorical fluency (animals)	26 (10; 8–44)	15.9 (6.4; 5–36)	≥21.9	<0.001
MCST criterion	3 (0; 3)^e^	2.5 (0.7; 1–3)^f^	≥1.8	<0.05
MCST series	6.4 (1.7; 3–8)^e^	4.4 (2.4; 1–7)^f^	-	<0.05
**Forward Digit Span**	6.5 (1.3; 4–9)	5 (1; 3–7)	≥4.3	<0.001
**Backward Digit Span**	4.4 (1.6; 2–8)	3.2 (0.7; 2–5)	≥2.7	<0.01
**Depression - MADRS**	11.6 (6.8; 3–27)^g^	16 (7.2; 4–29)^h^	<15	<0.05

HD1: patients at Stage 1, HD2: patients at Stage 2; TFC: Total Functional Capacity; UHDRS: United Huntington Disease Rating Scale; MDRS: Mattis Dementia Rating Scale; FCSRT: Free and Cued Selective Reminding Test; RAVLT: Rey Auditory Verbal Learning Task; TMT-A; Trail Making Test form A; TMT-B: Trail Making Test form B; Stroop C/W: Stroop Color Interference Test; MCST: Modified Card Sorting Test; MADRS: Montgomery and Åsberg Depression Rating Scale; ns: non significant.

a : 17/25;

b : 18/21;

c : 19/25;

d : 14/21;

e : 15/25;

f : 15/21;

g : 24/25;

h : 20/21.

* cut-off are provided according to [21].

### Subjective evaluation of memory by patients

**Figure 1:**
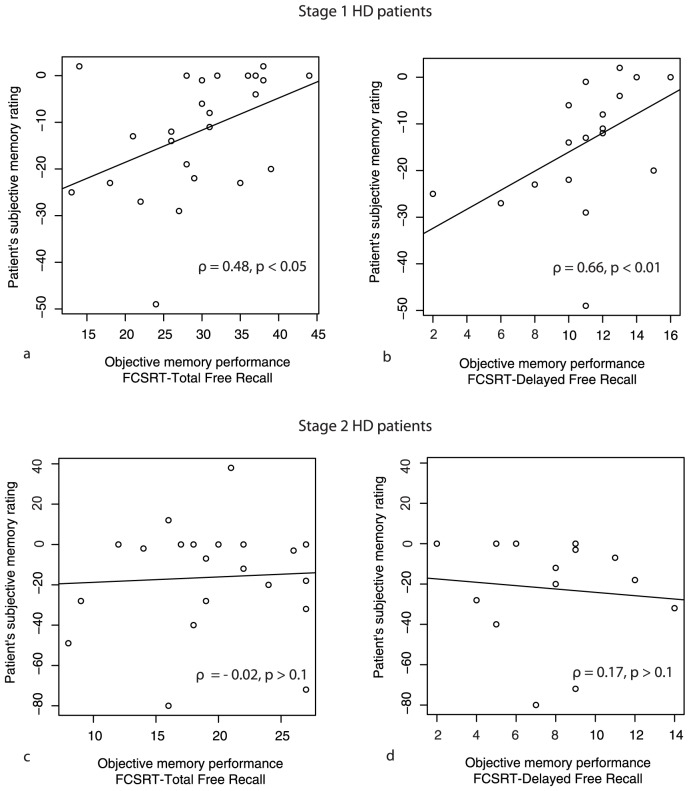
Awareness of memory deficits in Stage 1 but not in Stage 2 patients. Spearman correlations between objective memory performance and patients' subjective memory rating at Stage 1 and Stage 2. FCSRT-TFR: Free and Cued Selective Reminding Test - Total Free Recall; FCSRT-DFR: Free and Cued Selective Reminding Test - Delayed Free Recall.

The subjective memory score was similar in HD1 (average score: −12.1±12.9) and in HD2 (average score: −16.2±27.5) for both the total scores and the subscores (t-test, t = 0.6, p = 0.5). In HD1, both the total score and the subscores on the subjective memory questionnaire correlated with the objective memory performance at the FCSRT but not with the recognition score of the RAVLT (Table 2 and Figure 1). In contrast, in HD2, there was no correlation between subjective memory evaluation and objective performance at the FCSRT. However, there was a positive correlation with their recognition score of the RAVLT (Table 2 and Figure 1). Subjective rating of memory dysfunction was similar in patients with and without cognitive impairment, as it was the case for patients with and without depression (t-test, p>0.1).

**Table 2:** Spearman correlations between subjective memory rating by HD patients and objective memory testing.

	Subjective Memory Rating													
	HD1							HD2						
	Total	Global	Attention	Retention	Recall	Remote	Meta	Total	Global	Attention	Retention	Recall	Remote	Meta
**FCSRT**														
Total Free Recall	**0.48** *	**0.49** *	**0.46** *	0.39	0.36	**0.47** *	**0.48** *	−0.02	−0.02	−0.07	−0.05	0.05	0.04	−0.09
Total Free & Cued Recall	0.34	0.21	0.24	0.35	0.24	**0.48** *	**0.57** **	−0.11	−0.26	−0.07	−0.08	−0.06	−0.03	−0.33
Recognition	0.26	0.13	0.18	0.27	0.15	0.32	**0.49** *	−0.01	0.03	−0.08	−0.06	0.18	−0.15	−0.05
Delayed Free Recall	**0.66** ** **** a	**0.55** *	**0.5** *	**0.61** **	**0.54** *	**0.47** *	**0.61** **	−0.17^b^	−0.16	−0.28	−0.24	−0.24	0.07	−0.03
Delayed Free & Cued Recall	0.31^a^	0.15	0.43	0.24	0.2	0.02	0.18	−0.36^b^	−0.39	−0.24	−0.4	−0.25	−0.26	−0.27
**RAVLT**														
Total Recall	0.18	0.23	0.33	0.09	0.01	0.23	0.32	−0.14	−0.08	−0.31	−0.26	−0.2	−0.1	−0.06
Recognition	0	0.09	−0.02	−0.02	−0.12	0.22	0.32	**0.45** *	0.42	0.33	0.31	0.38	**0.44** *	**0.54** *

HD1: patients at Stage 1, HD2: patients at Stage 2; FCSRT: Free and Cued Selective Reminding Test; RAVLT: Rey Auditory Verbal Learning Task. Total: sum of all responses; Global: global evaluation of memory; Remote: remote memory for personal events; Meta: metamemory. Only the values of Spearman's ρ are reported.

* p<0.05;

** p<0.01;

a : 19/25;

b : 14/21.

### Patients versus proxies subjective ratings

**Figure 2:**
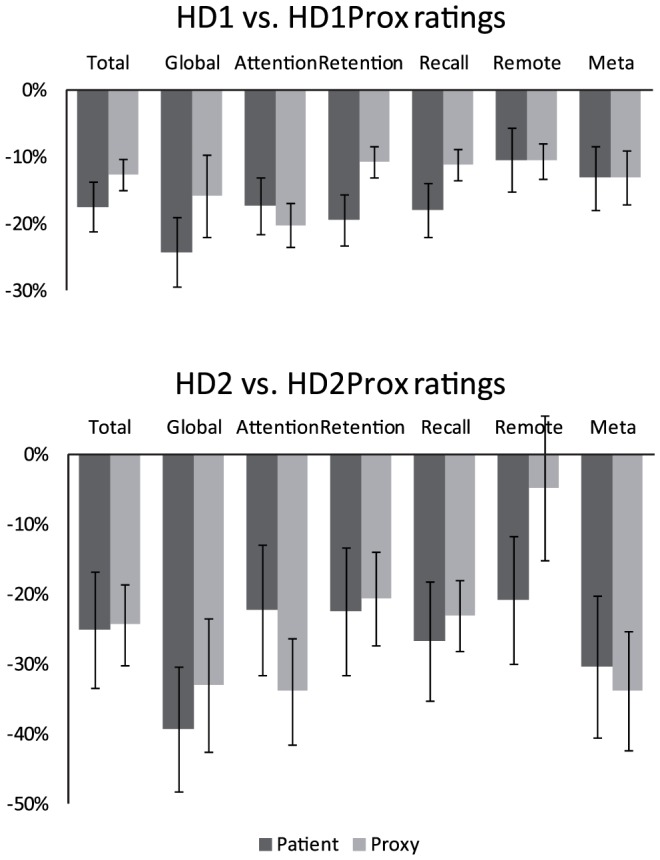
Subjective memory evaluation by patients and proxies at Stage 1 and Stage 2. HD1: patients at Stage 1, HD2: patients at Stage 2; HD1Prox: proxies of patients at Stage 1; HD2Prox: proxies of patients at Stage 2; Scores are transformed into the percentage of the maximal score for the evaluation of memory deficit. Higher percentages correspond to higher complaints. Error bars represent standard errors. Total: sum of all responses; Global: global evaluation of memory; Remote: remote memory for personal events; Meta: metamemory.

The subgroup of 33 patients who had a subjective evaluation by their proxies is representative of the above group of 46 patients in terms of demography, functional decline and neuropsychological performance (t-test, all p>0.5). In this subgroup, despite similar average subjective rating in HD1 and HD2 (HD1 −14 (±13.1); HD2 −20.1 (±24.8); t-test, t = 0.8; p = 0.4), scoring of memory impairment was more severe in HD2Prox than in HD1Prox (HD1Prox −10.1 (±8.2); HD2Prox −19.5 (±17.3); t-test, t = 1.9, p = 0.08). Patients and proxies' subjective memory ratings correlated (ρ = 0.4, p<0.05), with higher agreement for global memory and metamemory subscores (Table 3 and Figure 2). Both patients' and proxies' subjective ratings correlated with FCSRT performance at Stage 1 (HD1 ρ = 0.65, HD1Prox ρ = 0.48), but not at Stage 2 (Table 4). Proxies' subjective rating correlated with functional decline both for HD1 and HD2 patients, and with depression only in HD2 patients (Table 4). Overall, the proxies' subjective ratings were similar for patients with and without cognitive impairment (t-test, p>0.1), but were more severe for patients with depression, in comparison to patients without depression (t-test, t = 2.8, p = 0.01).

**Table 3:** Scale content and patient-proxy agreement for individual items (N = 33).

Item content	Patient-proxy agreement		
	All	HD1-HD1Prox	HD2-HD2Prox
**Global memory**	**0.57** ***	**0.5** *	**0.55** *
my memory is	**0.51** **	0.37	**0.57** *
the judgment of my relatives about my memory is	**0.59** ***	**0.51** *	**0.7** **
**Attention**	**0.35** *	0.36	0.4
my ability to make sense out of what people explain to me is	0.09	0.02	0.04
my ability to pay attention to what goes on around me is	0.34	0.28	0.45
my ability to follow what people are saying is	0.18	0.05	0.36
my general alertness to things happening around me	**0.4** *	0.38	0.48
my ability to answer these questions is	0.17	0.38	−0.08
**Retention**	**0.44** *	0.36	0.55
my ability to hold in my memory things that I have learned is	0.11	0.27	0.06
my ability to remember the names and faces of people is	**0.41** *	**0.46** *	0.47
my ability to remember what I read is	**0.35** *	**0.46** *	0.29
my ability to remember what I watch on television is	0.33	−0.03	**0.58** *
my ability to remember what I was doing after few minutes is	0.16	0.03	0.28
**Recall**	0.34	0.43	0.35
my ability to search into my mind and recall names is	**0.45** **	**0.62** **	0.3
my ability to recall things when I really try is	−0.07	−0.29	0.18
my tendency for a memory to be on the tip of the tongue is	**0.46** **	**0.51** *	**0.54** *
my ability to recall what happened a few minutes ago is	**0.45** **	0.36	**0.55** *
**Remote memory for personal events**	0.29	0.12	0.53
my ability to remember things that happened>one year ago is	0.16	−0.1	0.36
my ability to recall things that happened a long time ago is	0.3	0.14	0.46
my ability to recall things that happened during childhood is	**0.35** *	0.31	0.41
**Metamemory**	**0.46** **	**0.5** *	0.48
my ability to remember this questionnaire in one month is	**0.46** **	**0.5** *	0.48

The item content corresponds to the item question of the HD patients. These items were modified for the proxy, replacing the pronoun “I” by “he” or “she”, as appropriate. HD1: patients at Stage 1, HD2: patients at Stage 2; HD1Prox: proxies of patients at Stage 1; HD2Prox: proxies of patients at Stage 2. Only the values of Spearman's ρ are reported.

* p<0.05;

** p<0.01;

*** p<0.001.

**Table 4:** Relationship of subjective memory ratings of patients and proxies to objective clinical measures.

	Stage 1		Stage 2	
	HD1	HD1Prox	HD2	HD2Prox
**Memory: FCSRT**				
Total Free Recall	**0.65** **	**0.48** *	0.25	0.09
Total Free & Cued Recall	0.36	0.4	0.08	−0.42
Recognition	0.27	0.02	0.03	0.13
Delayed Free Recall	**0.62** *	0.32	0.27	−0.02
Delayed Free & Cued Recall	0.33	−0.05	−0.11	−0.57
**Memory: RAVLT**				
Total Recall	0.4	0.19	−0.24	−0.03
Recognition	0.35	0.12	0.46	**0.58** *
**Global cognition: MDRS**				
Total	0.37	−0.02	0.01	0.45
Memory	0.19	−0.11	−0.06	0.15
**Executive functions**				
MCST-Series	0.18	−0.04	**−0.74** *	−0.32
MCST-Criterion	NA§	NA§	**−0.88** **	−0.42
TMT A (time)	−0.09	0.17	0.19	−0.07
TMT B (time)	−0.07	0.08	0.00	−0.26
TMT B (points)	−0.23	0.11	−0.15	0.26
Stroop C/W	0.43	0.18	−0.06	0.23
Forward Digit Span	0.41	−0.14	0.27	0.22
Backward Digit Span	0.21	−0.06	−0.17	−0.15
Literal Fluency	0.04	0.01	0.04	0.41
Categorical Fluency	0.12	0.2	0.2	0.44
**Functional decline: TFC**	0.43	**0.58** **	0.1	**0.73** **
**Disease duration**	0.13	0.05	0.13	−0.19
**Depression: MADRS**	−0.13	0.05	−0.25	**−0.74** **

HD1: patients at Stage 1, HD2: patients at Stage 2; HD1Prox: proxies of patients at Stage 1; HD2Prox: proxies of patients at Stage 2; FCSRT: Free and Cued Selective Reminding Test; RAVLT: Rey Auditory Verbal Learning Task; TFC: Total Functional Capacity; MDRS: Mattis Dementia Rating Scale; TMT-A; Trail Making Test form A; TMT-B: Trail Making Test form B; Stroop C/W: Stroop Color Interference Test; MCST: Modified Card Sorting Test; MADRS: Montgomery and Åsberg Depression Rating Scale. Only the values of Spearman's ρ are reported.

* p<0.05;

** p<0.01;

§ no correlation because of a ceiling effect: all patients performed normally. Note that due to a low number of observations for some tasks, Spearman correlations did not reach significance, despite relatively high coefficients.

### Awareness indexes

The mean Δ(subjHD-subjProx) index was −2.5 (±15), and it was similar for HD1 and HD2 (−3.9 (±12.3) and −0.6 (±18.4), respectively; t-test, t = 0.6, p = 0.6). According to this index, only 2 patients showed severe unawareness for memory deficits, 11 were mild impaired and 20 patients were fully spared [10]. The Δ(subjHD-subjProx) index did not correlate with clinical variables related to the disease evolution (Table 5).

**Table 5:** Relationship between awareness indexes and clinical variables.

	Awareness indexes		
	Δ(subjHD-subjProx)	Δ(subjHD-objHD)	Δ(subjProx-objHD)
**Age**	−0.04	−0.06	−0.07
**Years of education**	−0.09	**−0.72** ***	**−0.54** **
**CAG repeats**	0.12	**0.48** **	0.34
**Disease duration**	0.15	0.41	0.08
**Functional Decline-TFC**	−0.12	**−0.49** **	−0.24
**UHDRS-Aptitude**	0.18	**0.54** **	0.32
**UHDRS-Motor**	0.15	**0.66** ***	**0.42** *
**UHDRS-Psychiatric**	−0.17	−0.1	0.01
**Depression - MADRS**	−0.03	0.11	0.01
**Global Cognitive Efficiency - MDRS**	0.13	**−0.49** **	**−0.43** *
**Memory**			
**FCSRT**			
Total Free Recall	0.22	**−0.46** **	**−0.49** **
Total Free & Cued Recall	0.16	**−0.36** *	**−0.43** *
Recognition	−0.06	**−0.5** **	**−0.38** *
Delayed Free Recall	0.13	**−0.5** *	**−0.36**
Delayed Free & Cued Recall	0.12	**−0.41** *	**−0.43** *
**RAVLT**			
Total Recall	0.01	**−0.65** ***	**−0.57** ***
Recognition	0.12	**−0.4** *	**−0.35** *
**Executive function**			
TMT A time	−0.05	0.3	0.19
TMT B time	0.03	**0.56** ***	**0.35** *
TMT B points	−0.12	**−0.4** *	−0.18
Literal fluency (P, M)	−0.02	**−0.42** *	−0.24
Categorical fluency (animals)	0.02	**−0.5** **	−0.3
MCST criterion	−0.4	**−0.69** ***	**−0.54** *
MCST series	−0.15	**−0.54** *	0.41
Stroop C/W	0.08	**−0.37** *	−0.32
**Forward Digit Span**	0.14	−0.18	−0.15
**Backward Digit Span**	−0.003	**−0.48** **	**−0.42** *

The Δ(subjHD-subjProx) index compares subjective ratings by patients and proxies. The Δ(subjHD-objHD) index compares subjective ratings by patients with objective memory performance. The Δ(subjProx-objHD) index compares subjective ratings by proxies and objective memory performance. HD1: patients at Stage 1, HD2: patients at Stage 2; HD1Prox: proxies of patients at Stage 1; HD2Prox: proxies of patients at Stage 2; FCSRT: Free and Cued Selective Reminding Test; RAVLT: Rey Auditory Verbal Learning Task; TFC: Total Functional Capacity; MDRS: Mattis Dementia Rating Scale; TMT-A; Trail Making Test form A; TMT-B: Trail Making Test form B; Stroop C/W: Stroop Color Interference Test; MCST: Modified Card Sorting Test; MADRS: Montgomery and Åsberg Depression Rating Scale. Only the values of Spearman's ρ are reported.

* p<0.05;

** p<0.01;

*** p<0.001.

**Figure 3:**
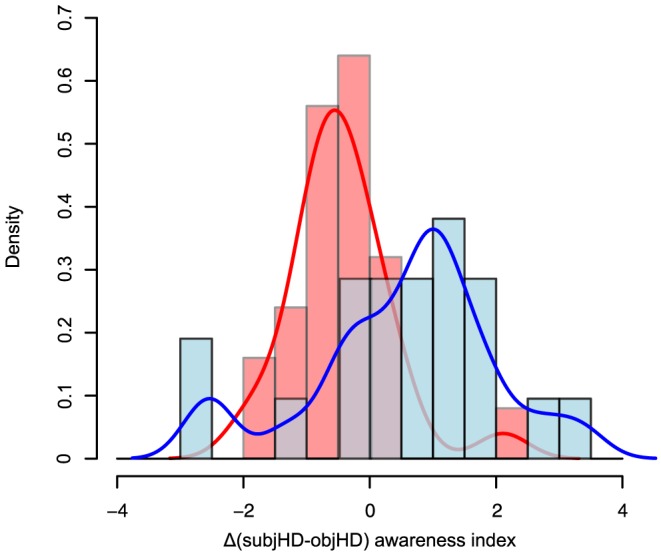
Distribution of memory awareness in HD 1 and HD2. Histograms and density curves represent the distributions of awareness of memory deficits according to the Δ(subjHD-objHD) index in HD1 (red) and HD2 (blue). Negative scores indicate preserved awareness, whereas positive scores indicate impaired awareness. HD1: patients at Stage 1, HD2: patients at Stage 2.

The Δ(subjHD-objHD) index differed according to the stage (HD1: −0.45 (±0.8); HD2: 0.54 (±1.5); t-test, t = −2.8, p = 0.009, Figure 3), whereas the Δ(subjProx-objHD) index did not (HD1: −0.3 (±0.8); HD2: 0.3 (±1.4); t-test, t = −1.5, p = 0.15). The Δ(subjHD-objHD) index correlated with almost all clinical variables linked to disease evolution, with the exception of behavioral ones. The number of clinical variables that correlated with the Δ(subjProx-objHD) index was lower than the number of those correlating with the Δ(subjHD-objHD) index (Table 5).

## Discussion

In this study, we assessed memory awareness in HD at an early stage. Our main finding is that HD1 are aware of their memory deficits, contrary to numerous reports of unawareness of deficits in this condition [2], [3], [4], [5], [6], [7], [8], [9]. Loss of awareness is associated with disease progression and is manifest in HD2, although even these patients acknowledge memory difficulties. In addition, proxies' evaluation of memory deficits in HD is less accurate than HD1 patients' evaluation about themselves. Thus, proxies' evaluation is not the more appropriate control to measure awareness of memory deficits in patients. Whereas proxies' evaluation relates to global decline, depression and memory deficits in patients, HD1 patients' evaluation specifically relates to the memory impairment.

In this cohort, although only 65% of HD patients showed cognitive impairment and 40% showed memory deficits, more than 90% (42 out of 46) subjectively identified a memory deficit. Similarly, 90% of proxies (30 out of 33) acknowledged this trouble. This suggests that HD patients and their proxies acknowledge memory troubles even when they are subtle. Surprisingly, there was no difference between subjective rating by HD1 and HD2, whereas HD2Prox subjective ratings were more severe than HD1Prox.

In order to test the accuracy of subjective ratings, we searched for correlations between subjective and objective evaluations. We used the objective performance at FCSRT and RAVLT in patients because these tests were able to detect a slight impairment of memory in the early stages of the disease and could gradually decline in more advanced stages. As expected, the FCSRT provided more sensitive results than the RAVLT in HD patients. Performance at the free recall subscores of the FCSRT did not show ceiling effect in HD1, in contrast to the one observed with total free recall subscore of the RAVLT (Table 1). HD1 subjective rating correlated with free recall FSCRT subscores, while HD2 subjective rating did not. This supports the hypothesis that HD1 properly acknowledge and quantify their memory deficits while HD2 do not (Tables 1, 2 and Figure 1). Presumably, the high performance on the RAVLT in HD1 explains the absence of a correlation between subjective memory ratings and RAVLT scores. In HD2, the lower performance in the RAVLT correlated with patients' subjective ratings which could suggest some residual capacity for evaluating memory deficits at Stage 2 (Table 2). However, such a correlation between subjective ratings of memory and RAVLT performance was not found in a recent study which included more advanced HD patients [9]. Finally, rating of the metamemory item of the subjective questionnaire correlated with FCSRT performance in HD1 and with RAVLT performance in HD2. This finding suggests that both HD1 and HD2 may succeed in a performance prediction test [34] in which the patients predict their future performance for a cognitive task (for example remembering a set of words) (Table 2). As a whole, the difference of correlations between subjective rating and objective performance according to the stage in our cohort of patients suggests that HD1 and HD2 behave differently regarding their memory awareness. However, because subjective memory evaluation is the same in HD1 and HD2, the differences in correlations could be driven by differences in memory scores (Table 1) and not by awareness *per se*. This could suggest either that: 1) both HD1 and HD2 acknowledge memory deficits without being able to properly quantify them, or 2) HD1 acknowledge and properly quantify their memory trouble, while HD2 only acknowledge it.

In order to disentangle both hypotheses, we used a contrast approach to compare subjective ratings and objective performance both in patients and proxies. The analysis of the Δ(subjHD-objHD) index supports the second hypothesis by showing spared awareness in HD1 (negative index) and impaired awareness in HD2 (positive index) (Figure 3). Accordingly, the Δ(subjHD-objHD) index correlated with almost all disease burden variables (including CAG repeats), except behavioral ones: the more severe is the disease the higher unawareness for memory deficits (Table 5). This indicates that unawareness for memory deficits in HD is related to disease severity [2], [3], [4], [5], [6], [7], [8], [9] and not to a depressive bias as in PD [12]. Noteworthy, lower education is associated with lower awareness of memory deficits in HD.

Proxies also report memory deficits in patients from the earliest stages of the disease (Figure 2). However, their estimation is not highly correlated with the patients' objective performance. Thus the index of subjective ratings and objective performance in patients Δ(subjHD-objHD) is a better measure of patients' memory awareness than the index which compares subjective ratings by HD patients and by proxies Δ(subjHD-subjProx). Indeed, the analysis of the Δ(subjHD-subjProx) index using the cut-off provided in Michon et al. [10] suggests the existence of severe unawareness for memory deficits in only 4% of the HD patients although 40% of them suffered from memory deficits. This subjective index seems better adapted to the pattern of AD - where unawareness was detected in 50% of the patients at mild or moderate stage and correlated with executive dysfunction [10] - in contrast to HD patients (Table 5). Noteworthy, we found similar results using the method of underestimation and overestimation scores already used in PD and HD [9], [12] to compare subjective ratings by patients and by proxies (data not shown). More specifically, HD1Prox judgment of patients' memory dysfunction is less accurate than the one of HD1, as shown by a weaker correlation between HD1Prox subjective rating and objective measures of memory of the FCSRT and by a stronger correlation between HD1Prox rating and functional decline (TFC) (Table 4). A reverse tendency is found for the comparison of subjective ratings by HD2 patients and HD2Prox, with more accurate ratings of memory deficits by HD2Prox, yet both functional decline (TFC) and depression (MADRS) also impinge on HD2Prox judgment (Table 4). Finally, the Δ(subjProx-objHD) index, which evaluates proxies' awareness of patients' memory deficits, does not differ between HD1Prox and HD2Prox, and is less correlated with disease severity markers than the Δ(subjHD-objHD) index (Table 5). This result is of importance, because the proxies' assessment is frequently used as a control to evaluate awareness of deficits in several neurodegenerative diseases (in AD [10], [11], PD [12] and HD [2], [3], [4], [6], [9]). However, the fact that HD is a genetic dominant disease with a long asymptomatic phase probably has specific emotional and psychological consequences on proxies' assessments. For example, proxies might experience guilt (associated with not being ill themselves), denial (of the apparition of the deficit), or even bitterness (for transmitting the disease to the children) [2]. In clinical practice, one should acknowledge this potential bias in proxies' judgments when testing the awareness of a particular deficit in HD.

The finding that HD1 are aware of their memory deficits seems to contradict previous reports according to which HD patients exhibit general and equivalent unawareness of behavioral, cognitive and motor symptoms [4], [5]. Although not denying that HD yields unawareness of a wide range of symptoms, our study raises the hypothesis that unawareness of different symptom domains occurs at different moments during the course of the disease. Accordingly, presymptomatic gene-carriers of HD are suspected to become less aware of their frontal behavioral symptoms when approaching the symptomatic phase of the disease [35], while unawareness of chorea [6], [7], [8] is probably present from the beginning of the symptomatic phase. At later stages of HD, one would expect to observe unawareness of various symptoms. If this hypothesis were verified, this would argue against the view that awareness of deficits should be examined as a general process in HD [5], [36], and would instead suggest a sequential impairment of distinct processes of awareness of specific deficits parallel to disease evolution [37], [38]. Future studies should assess intra-subject comparisons of awareness of different symptoms (such as motor or behavioral symptoms), with objective and subjective measures in order to understand the time course of the appearance of the lack of awareness in each domain and to delineate the usefulness of this kind of questionnaire at each stage and for each domain.

The comparison with other degenerative disorders in which awareness of memory deficits has been examined could provide information about the neural basis of our results in HD. The fact that both HD1 and PD [12] patients remain aware of their memory deficits may be related to predominant subcortical degenerative process in these populations. Indeed, although several studies point out that HD patients show cortical atrophy even at the early stages of the disease [39], [40], striatal atrophy remains the earliest neuroanatomical signature of disease evolution in HD [15]. Conversely, the lack of awareness of memory deficit in HD2 [9] and AD [10] might be associated with more widespread cortical atrophy. Accordingly, AD patients showing unawareness of their deficits showed a reduced functional recruitment of the cingulofrontal and parietotemporal regions [41]. Precise correlations with neuroanatomy are now needed to determine if this result also applies to HD.

To conclude, our results show that HD patients at early stage remain aware of their memory deficits, contrary to previous reports suggesting unawareness for cognitive deficits and behavioral disturbances in this condition. Hence, unawareness in HD is not a general and uniform process but rather a domain-specific one. We suggest that in order to better evaluate awareness of a specific deficit in HD, it may be necessary to compare a patient's subjective evaluation with a patient's objective performance and not only to rely on a proxy's judgment. Future studies are needed to explore the reliability of auto-questionnaires to evaluate HD patients' deficits (including memory) at early stages of the disease.
